# Changes in concentration performance and alternating attention after short-term virtual reality training in E-athletes: a pilot study

**DOI:** 10.1038/s41598-024-59539-w

**Published:** 2024-04-17

**Authors:** Maciej Lachowicz, Alina Żurek, Dariusz Jamro, Anna Serweta-Pawlik, Grzegorz Żurek

**Affiliations:** 1 Department of Biostructure, Wroclaw University of Health and Sport Sciences, 51-612 Wrocław, Poland; 2 Institute of Psychology, University of Wrocław, 50-137 Wrocław, Poland; 3 Department of Physical Education and Sport, General Tadeusz Kosciuszko Military University of Land Forces, 51-147 Wrocław, Poland; 4 Department of Occupational Therapy, Wroclaw University of Health and Sport Sciences, 51-612 Wrocław, Poland

**Keywords:** Attention, Learning and memory

## Abstract

In the dynamic landscape of e-sports, where intense competitive gaming demands high cognitive abilities, concentration performance and alternating attention play a pivotal role. E-sports encompass diverse genres, each requiring specific cognitive functions. Maintaining unwavering focus is crucial, as split-second decisions can determine victory. The study explores the potential of Virtual Reality (VR) training to enhance concentration performance and alternating attention, shedding light on the importance and possibilities of optimizing cognitive abilities for e-athletes. VR training emerges as a promising intervention, offering immersive environments for cognitive exercises. The study investigates the impact of VR training on concentration performance and alternating attention in amateur e-athletes, utilizing standardized tests. A randomized controlled trial with 66 participants reveals significant improvements in the VR training group, highlighting the adaptability and plasticity of cognitive processes. The findings suggest that VR training can enhance concentration abilities, providing valuable insights for e-sports and potentially extending to other fields requiring sustained attention and rapid task-switching. The study underscores the convergence of cognitive psychology, neuroscience, and VR technology, paving the way for innovative training methodologies and advancements in e-sports performance.

## Introduction

In the dynamic and rapidly evolving realm of e-sports, where players engage in intense competitive gaming across various genres and platforms, the significance of cognitive abilities in und. Among them, the significant role of concentration^1^ performance and alternating attention^2^ is indicated. E-sports competitions often involve complex strategic gameplay, demanding high levels of cognitive engagement, rapid decision-making, and precise motor responses. As the popularity and competitiveness of e-sports soar worldwide, the task to optimize the cognitive performance of e-athletes becomes paramount^3^. The competitive nature of e-sports magnifies the importance of cognitive functions (CF)^4^. In high-stakes tournaments and team-based competitions, e-athletes must synergistically collaborate with their teammates, communicate effectively, and synchronize their actions. Strong concentration performance and alternating attention among team members contribute to cohesive teamwork, synchronized decision-making, and strategic coordination^5^. By improving various CF, e-athletes can elevate their individual performance while bolstering the overall synergy and effectiveness of their team. Moreover, the multidimensionality of e-sports necessitates a diverse set of CF depending on the game genre. E-sports encompasses an extensive array of game types, each presenting its unique cognitive demands. For instance, within the e-sports realm, we find various genres such as first-person shooters (FPS) e.g. Counter Strike: Global Offensive, real-time strategy (RTS) games e.g. Starcraft or Age of Empires, multiplayer online battle arena (MOBA) games e.g. League of Legends, sports simulations, and fighting games. Each of these genres requires e-athletes to exhibit distinct CF and adapt their strategies accordingly. For instance, FPS games demand rapid reaction times, pinpoint accuracy, and acute situational awareness^6^. In contrast, MOBA games emphasize strategic thinking, resource management, and effective coordination with teammates^1^. RTS games call for quick decision-making, long-term planning, and the ability to multitask seamlessly^7^. Each game genre places unique cognitive demands on e-athletes, making it essential to develop and hone a range of cognitive abilities tailored to the specific gameplay requirements. Concentration performance is particularly crucial in e-sports, as players must maintain unwavering focus and attention for extended durations^8^. Whether it is tracking opponents’ movements, anticipating game events, or coordinating team strategies, e-athletes must sustain their attentional resources amidst an abundance of distractions and sensory stimuli^9^. The capacity to sustain unwavering focus and undivided attention throughout training and competitive events is critical for achieving optimal results. In the realm of e-sport, split-second decisions, rapid reactions, and precise motor responses often delineate the difference between victory and defeat^6^. The ability to remain fully engaged and immersed in the game is pivotal for achieving optimal performance. Such a heightened level of engagement necessitates not only robust mental endurance but also the capacity to endure extended intervals of both mental and physical exertion, often characterized by prolonged periods of sedentary activity. E-athletes frequently, especially during important tournaments, confront the demand to sustain a heightened state of cognitive attentiveness and acuity throughout protracted sessions, which may extend over several hours or even more. Even momentary lapses in concentration can result in missed opportunities, strategic missteps, or vulnerability to opponents’ attacks^10^. Another important factor in successful e-sports performance is the alternating attention (AA), which encompasses the capability to flexibly shift focus between multiple tasks, stimuli, or game objectives^11^. E-sport often requires e-athletes to rapidly switch between different CF, such as monitoring various game elements simultaneously, making real-time decisions, and adjusting strategies^12^. Effective task-switching and divided attention enable e-athletes to juggle multiple gameplay aspects, adapt to evolving situations, and allocate their cognitive resources efficiently. Proficient alternating attention allows e-athletes to seamlessly transition between different gameplay elements, maintain situational awareness, and respond swiftly to emerging challenges or opportunities. Neuroscientific studies have highlighted the neural underpinnings of concentration performance and alternating attention. These CF rely on intricate networks within the brain, involving regions such as the prefrontal cortex, parietal cortex, and sensory processing areas^13,14^. Attentional control mechanisms enable the regulation of attentional resources, allowing individuals to prioritize relevant information and filter out distractions. Executive functions, including cognitive flexibility and working memory, contribute to effective task-switching and the seamless transition between different cognitive demands in various settings^15,16^. Additionally, sensory processing mechanisms facilitate the rapid and accurate perception and interpretation of game-related stimuli. Recognizing the critical role of cognitive abilities in e-sports, the task to enhance these skills through targeted training interventions has gained traction. One such training intervention is the application of virtual reality (VR), which, due to its immersive character, strongly engages the participant in this world and is at the same time a fairly natural environment^17^. VR training has emerged as a promising approach, leveraging immersive environments, realistic simulations, and interactive feedback to engage e-athletes in focused cognitive exercises. VR platforms offer a novel and engaging medium to develop and refine concentration performance, alternating attention, and other cognitive skills, providing a unique opportunity for e-athletes to train in an environment that closely mirrors the challenges and complexities of competitive gaming^18,19^. Furthermore, it’s important to note that VR’s applications extend beyond e-sports training. VR can also serve as a valuable tool in various other domains, such as assisting in therapeutic interventions^20^ or as a platform for physical activity^21^. Interestingly, not only VR-based interventions but also video games have found utility as tools for enhancing concentration performance and executive functions in various populations, including patients with mild cognitive impairment^22^ and individuals affected by ADHD^23^. The utilization of cognitive training through video games has garnered significant attention within both clinical and non-clinical contexts, emerging as a promising avenue for enhancing various CF Notable studies by Seçer and Satyen ^24^, Dye et al. ^25^, and Blacker et al. ^26^ have underscored its efficacy in augmenting reaction time, processing speed, and visual working memory, respectively. A systematic review conducted by Pallavicini et al. ^27^ has provided compelling evidence regarding the beneficial impacts of video game training on cognitive and emotional skills among young adults. Furthermore, mentioned investigation demonstrated the effectiveness of both non-commercial and commercial video games, including commercial brain-training programs, in enhancing cognitive abilities. Important for our study, is to highlight that methodical evaluations comparing traditional video gaming methodologies with VR gaming, inclusive of exergames, reveal a significantly higher level of cognitive stimulation when employing the latter^28^. This evidence positions VR as a superior instrument for cognitive development. The adoption of VR for cognitive training, exemplified by the use of "Beat Saber," which qualifies as a moderate-intensity physical exercise^29^, gains additional validation from the documented association between physical fitness levels and the augmentation of concentration and attention capabilities^30^. Moreover, given that VR encompasses a multisensory environment, it holds potential as a catalyst for comprehensive brain stimulation. While direct scientific evidence supporting the efficacy of VR-based stimulation is still emerging, the effectiveness of multisensory stimulation is well-documented. Notably, Cheng et al. ^31^ have highlighted its benefits in the context of neurocognitive disorders, and recent findings by Helbling et al. ^32^ further corroborate its utility in mitigating cognitive impairments. By leveraging the power of VR, e-athletes can potentially enhance their concentration performance and alternating attention abilities, thereby improving their overall performance in competitive gaming^33^. This convergence of cognitive psychology, neuroscience, and VR technology opens new horizons for investigating and optimizing the cognitive potential of e-athletes, paving the way for innovative training methodologies and advancements in the field of e-sports performance enhancement. While previous studies have explored the benefits of VR training in cognitive domains, such as memory^34,35^, decision-making^36^, spatial awareness^37^, visuospatial memory^38^ and other CF, to our knowledge, there have been no studies to date regarding VR training of concentration performance and alternating attention in amateur e-athletes. Therefore, this study aims to bridge this gap by investigating the impact of VR training on these cognitive abilities. By exploring the potential of VR training, we aim to provide valuable insights into the development of cognitive training strategies for e-sports. Presented study may have broader implications for the application of VR training in other fields like motor sports or medical training that require sustained attention and rapid task-switching abilities.

## Results

Obtained results of both Cognitrone (COG) and Color Trail Test (CTT) tests are shown as mean values ± standard deviation in Tables 1 and 2. The statistical analysis conducted in this study employed 2 × 3 × 6 mixed model Analysis of Variance (ANOVA), results shown in Table 3, which revealed significant main effects of group (F = 7.116, p = 0.010, η^2^p = 0.125, λ = 7.116), measurement (F = 1344.137, p < 0.000001, η^2^p = 0.964, λ = 6720.687) and time (F = 25.164, p < 0.001, η^2^p = 0.335, λ = 50.329). Interactions: measurement x group (F = 5.419, p < 0.0001, η^2^p = 0.098, λ = 27.093), time x group (F = 9.899, p < 0.001, η^2^p = 0.165, λ = 19,799), measurement x time (F = 9.168, p < 0.001, η^2^p = 0.155, λ = 91.676) and group x measurement x time (F = 5.438, p < 0.001, η^2^p = 0.098, λ = 54.383) were also significant.

**Table 1:** Obtained COG test results presented as mean values ± standard deviation.

Group	Variable		Pre-test Mean ± SD	Post-test Mean ± SD	Δ% pre-post	Follow-up-test Mean ± SD	Δ% pre-follow-up	Δ% post-follow-up
E	Reactions	CR	64.42 ± 5.82	71.42 ± 3.98	+ 10.87%	71.5 ± 5.30	+ 10.99%	+ 0.11%
		IR	13.92 ± 6.27	10.69 ± 5.34	− 23.20%	9.35 ± 4.04	− 32.83%	− 12.54%
		OR	15.15 ± 5.57	8.57 ± 3.98	− 43.43%	8.5 ± 5.30	− 43.89%	− 0.82%
C	Reactions	CR	64.65 ± 6.93	65.53 ± 6.91	+ 1.36%	64.96 ± 7.27	+ 0.48%	− 0.87%
		IR	16.19 ± 6.96	13.38 ± 7.49	− 17.36%	14.08 ± 7.51	− 13.03%	+ 5.23%
		OR	15.35 ± 6.93	14.46 ± 6.91	− 5.80%	15.08 ± 7.28	− 1.76%	+ 4.29%

E—experimental, C—control, CR—correct reactions, IR—incorrect reactions, OR—omitted reactions.

**Table 2:** Obtained CTT test results presented as mean values ± standard deviation.

Group	Pre-test			Post-test			Δ% Pre–post	Follow-up-test			Δ% pre-follow-up	Δ% post-follow-up
	CTT-1	CTT-2	Int I	CTT-1	CTT-2	Int I		CTT-1	CTT-2	Int I		
E	33.51 ± 3.6	63.54 ± 5.41	23.53 ± 8.35	24.21 ± 2.15	50.54 ± 2.99	24.23 ± 8.69	CTT-1: − 27.75%	25.23 ± 2.31	51.31 ± 4.36	22.03 ± 9.31	CTT-1: − 24.71%	CTT-1: + 4.21%
							CTT-2: − 20.46%				CTT-2: − 19.25%	CTT-2: + 1.52%
							Int I: + 2.97%				Int I: − 6.37%	Int I: − 9.08%
C	32.31 ± 3.69	61.36 ± 4.76	26.05 ± 8.25	29.37 ± 2.53	59.86 ± 3.61	28.31 ± 9.01	CTT-1: − 9.10%	30.76 ± 2.557	59.32 ± 3.33	24.29 ± 8.06	CTT-1: − 4.80%	CTT-1: + 4.73%
							CTT-2: − 2.44%				CTT-2: − 3.32%	CTT-2: − 0.90%
							Int I: + 8.68%				Int I: − 6.76%	Int I: − 14.20%

**Table 3:** Results of mixed model ANOVA for main effects and interactions.

Statistical factor	Sum of squares	DF	Mean square	F	p-value	η^2^p	λ
Group	872.032	1	872.032	7.116	0.010	0.125	7.116
Measurement	558,493.302	5	111,698.660	1344.137	< 0.000001	0.964	6720.687
Time	1322.735	2	661.367	25.164	< 0.001	0.335	50.329
Measurement × Group	2251.430	5	450.286	5.419	< 0.0001	0.098	27.093
Time × Group	520.341	2	260.170	9.899	< 0.001	0.165	19.799
Measurement × Time	2798.591	10	279.859	9.168	< 0.001	0.155	91.676
Group × Measurement × Time	1660.163	10	166.016	5.438	< 0.001	0.098	54.383

Following step of analysis was searching for significant within-groups changes, presented in Table 4. In the E group, the COG test results indicated significant improvements after the intervention. Correct Reactions (CR) showed a substantial increase, with the analysis yielding a p-value of less than 0.001, an F-value of 18.834, reflecting a large effect size (η^2^p = 0.435) and a λ of 37.668. Incorrect Reactions (IR) and omitted reactions (OR) both exhibited notable decreases, with p-values of 0.002 and less than 0.001, F-values of 7.088 and 16.577, effect sizes of η^2^p = 0.224 and 0.404, and λ of 14.175 and 33.153, respectively. These results demonstrate the intervention's robust impact on enhancing the accuracy and reducing errors in cognitive processing. Additionally, for the CTT, the E group showed significant improvements in both CTT-1 and CTT-2. The results for CTT-1 revealed an F-value of 13.684, a p-value of less than 0.001, an effect size of η^2^p = 0.358, and a λ = 27.369. For CTT-2, the F-value was 15.893, with a p-value of less than 0.001, an effect size of η^2^p = 0.393, and λ = 31,787. Int I did not show a significant change, with a p-value of 0.264. Contrastingly, the C group did not, except for a minor significance in IR (p-value of 0.044), demonstrate statistically significant changes across the same variables. The p-values were above the threshold of significance (CR: p = 0.755, OR: p = 0.752), suggesting that the cognitive function remained stable over time without intervention. The effect sizes were correspondingly low (η^2^p ≤ 0.121, λ ≤ 6.714), reinforcing the lack of significant cognitive change within this group. For the C group's CTT results, neither CTT-1 nor CTT-2 showed significant changes post-intervention, with p-values of 0.377 and 0.59, respectively. The effect sizes were (η^2^p ≤ 0.039, λ ≤ 1.992) below the threshold that might indicate a reliable detection of a true effect, if present.

**Table 4:** Results of mixed model ANOVA for within-groups changes.

Group	Test	Variable	p-value	F-value	η^2^p	λ
E	COG	CR	< 0.001	18.834	0.435	37.668
		IR	0.002	7.088	0.224	14.175
		OR	< 0.001	16.577	0.404	33.153
	CTT	CTT-1	< 0.001	13.684	0.358	27.369
		CTT-2	< 0.001	15.893	0.393	31.787
		Int I	0.264	1.368	0.53	2.737
C	COG	CR	0.755	0.283	0.011	0.567
		IR	0.044	3.357	0.121	6.714
		OR	0.752	0.286	0.012	0.573
	CTT	CTT-1	0.377	0.996	0.039	1.992
		CTT-2	0.590	0.533	0.021	1.066
		Int I	0.614	0.492	0.020	0.984

E—experimental group, C—control group, CR—correct reactions, IR—incorrect reactions, OR—omitted reactions.

Following the ANOVA with Bonferroni post-hoc analysis and Cohen's D calculations, of which results are presented in table 5, new findings emerged. For group E, CR significantly improved from pre-test to post-test (p < 0.001, Cohen's d = − 1.40) and from pre-test to follow-up-test (p < 0.001, Cohen's d = − 1.27), indicating a large effect and sustained improvement, though no significant change was noted from post-test to follow-up-test (p = 1.00). IR saw a mild improvement from pre-test to post-test (p = 0.02, Cohen's d = 0.55) and remained better at follow-up (p = 0.01, Cohen's d = 0.87). OR in group E exhibited significant enhancement at both intervals (p < 0.001), with large effects (Cohen's d = 1.36 and 1.22). Both CTT1 and CTT2 showed substantial improvements, with high Cohen's d values (CTT1: p < 0.001, Cohen's d = 3.14; CTT2: p < 0.001, Cohen's d = 2.97) and remained high at follow-up (CTT1: p < 0.001, Cohen's d = 2.74; CTT2: p < 0.001, Cohen's d = 2.49). However, Int-I in group E showed no significant changes at any point (p-values around 0.43–1.00, Cohen's d around − 0.08 to 0.17). For group C, only IR was measured, displaying no significant change at any testing time (p = 0.44 from pre-test to post-test, Cohen's d = 0.39; p = 0.39 from pre-test to follow-up-test, Cohen's d = 0.29), indicating a stable performance.

**Table 5:** Results of Bonferroni test, with additional Cohen’s d values, for within-groups comparisons.

Group	Variable	Pre-test vs post-test		Post-test vs follow-up-test		Pre-test vs follow-up-test	
		Bonferroni test p-value	Cohen's d value	Bonferroni test p-value	Cohen's d value	Bonferroni test p-value	Cohen's d value
E	CR	< 0.001	-1.40	1.00	-0.02	< 0.001	-1.27
	IR	0.02	0.55	1.00	0.28	0.01	0.87
	OR	< 0.001	1.36	1.00	0.01	< 0.001	1.22
	CTT-1	< 0.001	3.14	0.81	-0.46	< 0.001	2.74
	CTT-2	< 0.001	2.97	1.00	0.21	< 0.001	2.49
	Int-I	0.43	-0.08	1.00	0.17	0.49	0.24
C	IR	0.44	0.39	1.00	-0.09	0.39	0.29

E—experimental group, CR—correct reactions, IR—incorrect reactions, OR—omitted reactions, Int-I—Interference Index.

In final, between-groups analysis, presented in Table 6, for the pre-test, no significant differences were observed across the variables, with p-values ranging from 0.22 to 0.91 and small Cohen's d values, indicating similar starting points for groups E and C. However, post-test and at follow-up, the differences became more pronounced. For CR and OR, significant improvements were found in the experimental group compared to the control group, with p-values less than 0.001 and Cohen's d values around 1.04 and − 1.04 respectively at post-test, and maintaining similar effect sizes at follow-up. In the case of IR, although the initial differences were not statistically significant, a notable change with a moderate effect size (Cohen's d = − 0.78) emerged by the follow-up-test, with a p-value of 0.01, indicating a delayed but significant improvement in group E. The Color Trails Test results (CTT1 and CTT2) also showed significant enhancements in group E over time, with large negative Cohen's d values, especially noticeable in post-tests and follow-ups (CTT1: p = 0.01, Cohen's d = − 2.20; CTT2: p = 0.00, Cohen's d = − 2.81), suggesting strong and sustained improvements. INT-I, however, remained consistently non-significant between groups across all time points, with the differences not reaching statistical significance, and Cohen's d values remained relatively low, indicating minimal between-group effect.

**Table 6:** Results of Bonferroni post-hoc test for between-groups E vs C comparisons.

Variable	Pre-test		Post-test		Follow-up-Test	
	Bonferroni test p-value	Cohen's d	Bonferroni test p-value	Cohen's d	Bonferroni test p-value	Cohen's d
CR	0.90	− 0.04	< 0.001	1.04	< 0.001	1.03
IR	0.22	− 0.34	0.14	− 0.41	0.01	− 0.78
OR	0.91	− 0.03	< 0.001	− 1.04	< 0.001	− 1.03
CTT-1	0.50	0.33	0.01	− 2.20	< 0.001	− 2.27
CTT-2	0.59	0.43	0.01	− 2.81	0.01	− 2.06
INT-I	0.73	− 0.30	0.79	− 0.46	0.32	− 0.26

CR—correct reactions, IR—incorrect reactions, OR—omitted reactions, Int I—Inteference Index.

## Discussion

This study aimed to investigate the effectiveness of VR training in improving concentration performance and alternating attention. The application of such a training as a strategy for enhancing CF, is consistent with prior research that has investigated the advantages of VR technology in various cognitive areas such as memory, decision-making, and visuospatial abilities, as demonstrated in research by Georgiev et al. ^39^, Maggio et al. ^40^, and Serweta-Pawlik et al. ^38^. The findings from this study offer additional confirmation of the advantages associated with VR cognitive training. More specifically, it underscores the potential of such training to enhance concentration performance and alternating attention, particularly within the context of e-athletes, an area that had not been extensively explored previously. By leveraging immersive VR environments and interactive training, the study demonstrates the potential of VR technology to train and enhance CF in a targeted and effective manner. These findings add concentration performance and alternating attention to the repertoire of trainable CF within the domain of VR-based interventions. It is a notable discovery as concentration performance holds paramount significance in the domain of both e-sport and traditional disciplines, playing a pivotal role in determining athletes’ performance outcomes^41,42^. The observed increase in mental efficiency, characterized by improved accuracy and fewer errors or omissions, highlights the importance of rapid decision-making abilities. This is particularly critical in the competitive world of e-sports, where split-second decisions can significantly impact a player’s performance. Furthermore, competitive e-sports often involve team-based competitions, where effective communication and synchronized actions among team members are essential to maintain attention and execute strategic game plans successfully^43^. In this context, the ability to avoid unintentional lapses in concentration becomes crucial, as such lapses can lead to missed opportunities, errors, and an overall decline in performance^8^. Consequently, a short-term VR stimulation strategy holds potential, not just within e-sports but also as a means to enhance cognitive performance and efficiency in broader contexts. Moreover, it suggests that such VR stimulation could have a positive influence on mental endurance, further contributing to overall cognitive robustness across diverse domains. The significant improvements observed in executive functions within just 8 days of intensive VR training can be attributed to a combination of factors, highlighting the adaptability and plasticity of cognitive processes. One significant contributor to these enhancements is the role of physical activity. Studies have shown that even short-term bouts of exercise can positively impact cognitive performance^44^. This aligns with our findings, where participants engaged in physically demanding VR training demonstrated significant gains in executive functions, however, it’s not just the physical aspect that contributes to these improvements. The immersive and interactive nature of VR gaming, coupled with the inherent fun and motivation it offers, creates a powerful cognitive stimulus. Participants are not merely exercising; they are mentally engaged and driven to excel within challenging virtual environments^45^. The perceived enjoyment and motivation during VR gaming can amplify cognitive outcomes. Furthermore, the competitive element present in both gaming and e-sports plays a significant role in boosting executive functions. Research has consistently shown that individuals engaged in competitive games exhibit more substantial gains in executive function skills compared to those in non-competitive or control conditions^46^. This suggests that the competitive nature of e-sports can intensify the cognitive benefits, demanding increased focus, rapid decision-making, and strategic thinking, all of which are essential components of executive functions. Moreover, the relationship between achievement levels and executive function skills is noteworthy. As participants progress to higher difficulty levels in the VR training, they engage in more intense physical and cognitive efforts^47^. This dual challenge, both physical and mental, creates a synergistic effect, resulting in more significant improvements in executive functions. Through dedicated improvement of their concentration abilities tailored to the demands of e-sports, e-athletes have the opportunity to heighten their cognitive sharpness, amplify their adaptability in rapidly changing scenarios, and unlock their comprehensive athletic capabilities. Importantly, this concept extends beyond the realm of e-sports, resonating with the broader population as concentration performance is a crucial aspect also in traditional sports^48^ and every-day activities^49^. Engaging in computer games not only necessitates concentration performance but also has the potential to enhance this cognitive skill^50^. In the process of interpreting our findings, it is pertinent to highlight the notable absence of comparable investigations into the domain of alternating attention and executive functions specifically within the framework of e-sports. The significance of executive functions in e-sports lies in their crucial role, necessitating players to fluidly shift their cognitive focus between multiple critical elements during gameplay. These elements encompass a player’s in-game decision-making, monitoring of opponent movements, effective team communication, strategic navigation within the virtual environment, and other cognitively demanding facets that mandate swift and flexible cognitive adjustments. When considering e-sports in the context of team-based activities, despite the limited body of research specifically addressing executive functions within e-sports, pertinent insights can be drawn from a study conducted by Da Waelle et al. ^51^. This study examined a group of children engaged in team sports and revealed that such collective endeavors not only demand but also foster the development of advanced executive functions, surpassing those required by individual-paced sports. Intriguingly, a differential pattern emerged for children participating in self-paced sports, where no discernible advantage in executive functioning was observed compared to non-athletic counterparts. Notably, the research has provoked an important question: can the behavioral strategies and cognitive advantages cultivated in traditional team sports be effectively transferred and applied to the domain of e-sports? This inquiry brings to the forefront the potential convergence of cognitive benefits across different forms of sports and competitive activities. A parallel investigation conducted by Verburgh et al. ^52^, comparing individuals in professional youth academies with their amateur counterparts, yielded noteworthy findings. The outcomes indicated that individuals in the amateur group exhibited comparatively lower executive functions when contrasted with their professionally affiliated counterparts. This disparity might imply that the requisite level of executive functions is contingent on the competitive intensity of the respective context. Considering the shared attributes of team-based dynamics and competitive nature between e-sports and traditional team sports, it can be inferred that the demand for heightened executive functions aligns in both domains. Consequently, the prospect of utilizing VR-based training emerges as a valuable resource not only for aspiring amateur e-athletes aiming for elevated performance standards but also for conventional athletes engaged in team-based sporting disciplines. Progressing beyond this, the integration of VR-based training represents an innovative and promising approach. This novel advancement holds potential as a source of optimism for individuals grappling with concentration-related disorders. Moreover, the evolution of VR hardware, encompassing advancements like haptic feedback devices and motion tracking systems, is poised to elevate sensory engagement and offer a more authentic interactive experience, thereby further amplifying the efficacy of cognitive interventions^53^. As researchers continue their exploration of VR’s potential within the realm of cognitive training and rehabilitation, it is anticipated that forthcoming interventions will seamlessly integrate VR technology as an essential component. This innovative approach is poised to foster optimal cognitive functioning by harnessing the capabilities of VR. Within the scope of athlete development, VR training stands as a promising instrument, delivering remarkably immersive and true-to-life simulations of sports scenarios. This immersive approach offers athletes a controlled and dynamic environment to hone and enhance their cognitive abilities. These meticulously designed VR training programs challenge athletes with intricate and unpredictable scenarios that replicate the demands of their specific sports, necessitating swift decision-making and finely-tuned motor responses.

## Methods

### Participants

**Figure 1:**
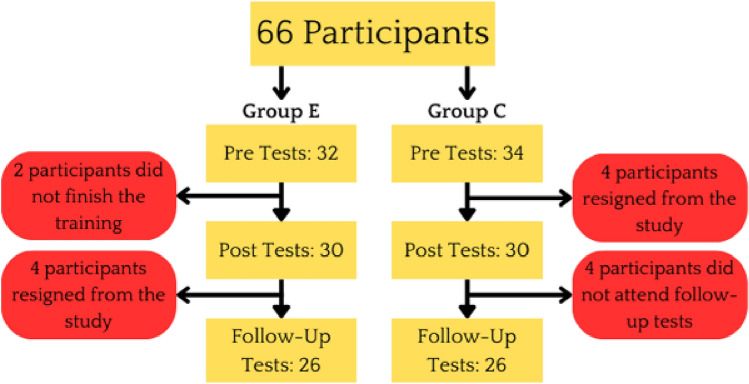
Number of participants in each stage of testing.

A total of 66 participants, comprising 43 men and 23 women aged 19–41 with an average age of 22.7 ± 0.66 years were recruited as subjects for this study. All participants affirmed their eligibility to meet the inclusion criterion, which required them to declare their status as active amateur e-athletes and confirm their non-involvement in professional e-sport competitions. A pre-study questionnaire was administered to gather pertinent information regarding the types of games most frequently played, average daily gaming time, e-sports experience, and age. The distribution of responses demonstrated similarity between the experimental group and control group, with MOBA games being the most prevalent, followed by FPS games. No statistically significant differences were observed between the E and C groups concerning average daily gaming time, e-sports experience, age, or initial levels of concentration performance and alternating attention. Using a simple 1:1 randomization method, the participants were, as shown in Fig. 1, assigned to either the experimental group consisting of 32 individuals who underwent training for eight consecutive weekdays, or the control group comprising 34 individuals. The randomization process ensured a balanced representation of both genders in both groups, blinded hypothesis process allowed to preserve objectivity of neuropsychological measurements The study experienced a dropout rate of 21.21 percent, only participants who completed the pre, post, and follow-up assessments were included in the final analysis. Individuals exhibiting neurological, visual, auditory, or motor disorders that impeded their ability to engage in VR training, as well as those who reported adverse side effects associated with virtual immersion, were, after randomized assignment to one of the groups, excluded from the study. The well-being of participants throughout the VR intervention was closely monitored using the Simulator Sickness Questionnaire, administered prior to and following each training session^54^. Recruitment for the study was conducted between November and December of 2022 at the Wrocław University of Health and Sport Sciences, Poland. Data collection took place in December 2022, and the study adhered to the ethical standards outlined in the Helsinki Declaration of 1964 and its subsequent revisions. Approval for the study’s methods and protocol (approval no. 19/2022) was obtained from the Research Ethics Committee of the Wroclaw University of Health and Sport Sciences. Prior to their participation, all subjects provided written, informed consent.

### Measurements

**Figure 2:**
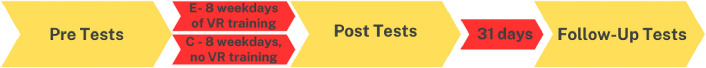
Flowchart of measurements.

As shown in Fig. 2, in the present study, assessments of concentration performance and alternating attention were conducted before the first training session and 30 min after completing the last training session for the E group. For the C group the pre- and post-tests were performed with an 8-day interval between assessments. Additionally, follow-up tests were conducted for both groups 31 days after the post-tests to evaluate any potential long-term effects of the training interventions

#### S4 Cognitrone/Vienna Test System (VTS)

To assess the level of concentration performance, the S4 Cognitrone test from the VTS was employed. VTS is a comprehensive and well-established platform for assessing cognitive and motor abilities, providing precise quantification of changes resulting from training interventions^55^. In the S4 Cognitrone test, respondents compare a given geometric figure with four other geometric figures and indicate whether the comparison figure is identical to any of the other options. Obtained results consisted of the number of correct responses, number of incorrect responses, and number of omitted responses.

#### Color Trail Test (CTT)

The study utilized the Color Trail Test to assess the focus of attention and executive functions. The CTT demonstrated commendable attributes of high validity and reliability, coupled with the simplicity of its tasks and procedures, thereby rendering it an exceedingly valuable diagnostic instrument within the domain of clinical psychology, particularly in the field of neuropsychology^56–58^. The test encompasses two inseparable components: CTT-1 and CTT-2, administered sequentially. CTT-1 measures visual search (VS), sustained attention (SA), and graphomotor ability through the analysis of performance time. On the other hand, CTT-2 provides supplementary insights into the subject’s capability for attentional divisibility, alternating attention (AA), sequential information processing and one’s own behaviour monitoring. Both segments of the test consist of colored circles, specifically pink and yellow, housing numerals. In CTT-1, pink circles display odd numbers, while yellow circles present even numbers. In CTT-2, each numeral is duplicated, once on a yellow background and once on a pink background. Before performing the actual tasks, the examinee is familiarized with the examples^58^. During the task, participants are required to meticulously connect the numerals in ascending order, adhering to the prescribed alternating color pattern and ensuring an uninterrupted sequence without lifting the pencil from the paper. The first part is usually performed faster than the second.

Subsequently, the accuracy and duration of this task are methodically recorded (CTT Professional Manual, PAR, accessed on 9th July 2023). The primary measure is the time, measured in seconds, to complete the actual task in each part (indicator: Test Time). Additional result which can be calculated is the Interference Index, allowing for the assessment of the impact of interfering factors on work speed. It is the difference between the time in CTT-2 and CTT-1, relativized to the time in CTT-1 (CTT-2 − CTT-1/CTT-1). The result is higher when there is a greater difference in the execution time of CTT tasks. A higher Interference Index indicates greater difficulties in coping with the challenges of the CTT-2 task. The test serves as a highly sensitive tool for detecting executive function disorders and attention processes affected by psychiatric and neurological causes. Due to the presence of equivalent versions A, B, C, and D, it can be successfully used in longitudinal studies. By utilizing these standardized tests, the study ensures objective and reliable measurements of CF across the different groups. This stage of the study, due to its strictly psychological character, was supervised by a certified psychologist.

### Study design

This was a two-group, randomized, single-blind, controlled trial comparing the VR training group to the control group. E group underwent eight immersive VR training sessions utilizing the game Beat Saber. These training sessions, with a duration of 15 minutes each, were conducted using the Valve Index VR headset. In contrast, the control group did not receive any VR training. Beat Saber is a VR game that involves wielding virtual lightsabers to slice through colorful blocks synchronized to the rhythm of the song. Each block corresponds to a specific color, and players must strike the blocks with the lightsaber of the corresponding color from the indicated, by an arrow on each block, direction. The game’s difficulty level gradually increased over time, progressing from the normal level to the expert level every 2 days. The selection of songs played during the training sessions was randomized to ensure variability and minimize bias. The game’s mechanics in Beat Saber base on concentration performance and alternating attention as participants must maintain focus, react quickly, and accurately strike the corresponding blocks following the visual and auditory cues provided. This training approach in Beat Saber offers a controlled environment to improve mentioned, relevant to e-sport performance, and CF. The randomized selection of songs further adds variation and adaptability to the training sessions, challenging participants’ ability to sustain attention and adapt to different rhythms and patterns.

### Statistical analysis

Statistical analyses were conducted in the Laboratory of Biostructure of the Wroclaw University of Health and Sport Sciences using IBM SPSS Statistics 27 software (https://www.ibm.com/products/spss-statistics). The laboratory is ISO 9001 certified, guaranteeing the adherence to high-quality standards. A significance threshold of p < 0.05 was established to ascertain statistically significant results. The distribution normality for the study variables was evaluated employing the Shapiro-Wilk test.

Initially, we calculated the means and standard deviations for the COG and CTT test outcomes. Subsequently, we carried out an analysis using a 2 × 3 × 6 mixed model ANOVA, examining the main effects and interactions related to group (experimental and control) as between-subjects factors, and measurement (CR, IR, OR, CTT-1, CTT-2, Int-I), and time (pre, post, follow-up) as within-subject factor. The Bonferroni post-hoc test, supplemented with Cohen’s d calculations, was utilized for final comparisons between and within-groups and time points to pinpoint significant differences accurately. This structured approach laid the groundwork for our study's statistical evaluations.

## Conclusion

Our study findings align with previous research that has showcased the promising advantages of VR technology in augmenting CF^38–40^. Specifically, the experimental group displayed discernible enhancements in both concentration performance and alternating attention metrics. These outcomes offer valuable insights into the capacity of customized training interventions to induce targeted enhancements in CF. The observed improvements in concentration performance and alternating attention highlight the potential of these interventions as beneficial preparatory tools for e-sports engagement. The results indicate that VR training may contribute to enhancements in these CF, which could, in turn, support the development of skills relevant to e-sports competitiveness. However, it is important to acknowledge that the direct impact of such training on e-sports performance itself remains to be fully explored. Future research is necessary to empirically establish the extent to which improvements in cognitive functions can translate into a competitive advantage in e-sports settings.

## Limitations

Despite our meticulous efforts to ensure a comprehensive research design, it is crucial to acknowledge certain inherent limitations in this study. One significant constraint is the absence of direct supervision of participants’ activities on weekends. Despite providing explicit instructions to adhere to their regular routines, the lack of continuous monitoring outside the controlled research environment introduces uncertainty regarding compliance. Another limitation concerns the reliance on self-report measures, specifically the questionnaires used to gather relevant data. Self-report instruments are susceptible to various biases, including social desirability and recall biases, which have the potential to distort participants’ responses, introducing inaccuracies into the collected data and affecting the overall validity of the study outcomes. Another consideration is. employing passive control group as it may complicate attributing observed effects directly to the intervention, and introduces variables related to participant engagement and external behaviors. Moreover, a potential confound arises due to the timing of the "post" measurement for the VR group, taken 30 min after their last session. This could lead to "carry-over" effects from the immediate cognitive and physical stimulation, affecting the interpretation of long-term cognitive benefits from VR training. Additionally, the question of measuring the influence of minimal interventions and determining the minimum intervention necessary to induce cognitive change remains an important area for further research. This inquiry is vital for establishing a more precise understanding of the efficacy and mechanisms of VR-based interventions in cognitive therapy. Furthermore, research aimed at directly measuring esports performance would provide definitive answers regarding the effects of cognitive interventions. Such studies would not only contribute to our understanding of how VR training can impact cognitive functions relevant to esports competitiveness but also help in tailoring cognitive interventions to meet the specific needs of esports athletes, ultimately enhancing their performance in competitive settings.

## Data Availability

Raw data presented in the study are not publicly available to preserve individuals’ privacy under the European General Data Protection Regulation. To access the data first author should be contacted.
